# Adjusting for under-identification of Aboriginal and/or Torres Strait Islander births in time series produced from birth records: Using record linkage of survey data and administrative data sources

**DOI:** 10.1186/1471-2288-12-90

**Published:** 2012-07-02

**Authors:** David Lawrence, Daniel Christensen, Francis Mitrou, Glenn Draper, Geoff Davis, Sybille McKeown, Daniel McAullay, Glenn Pearson, Stephen R Zubrick

**Affiliations:** 1 Telethon Institute for Child Health Research, Centre for Child Health Research, The University of Western Australia, P.O. Box 855, West Perth, WA, 6872, Australia; 2 Australian Bureau of Statistics, GPO Box K881, Perth, WA, 6842, Australia; 3 Department of Health, Government of Western Australia, Perth, Australia; 4 Australian Bureau of Statistics, PO Box 10, Belconnen, ACT, 2614, Australia; 5 Kurongkurl Katitjin, Centre for Indigenous Australian Education and Research, Edith Cowan University, 2 Bradford Street, Mount Lawley, WA, 6050, Australia

## Abstract

**Background:**

Statistical time series derived from administrative data sets form key indicators in measuring progress in addressing disadvantage in Aboriginal and Torres Strait Islander populations in Australia. However, inconsistencies in the reporting of Indigenous status can cause difficulties in producing reliable indicators. External data sources, such as survey data, provide a means of assessing the consistency of administrative data and may be used to adjust statistics based on administrative data sources.

**Methods:**

We used record linkage between a large-scale survey (the Western Australian Aboriginal Child Health Survey), and two administrative data sources (the Western Australia (WA) Register of Births and the WA Midwives’ Notification System) to compare the degree of consistency in determining Indigenous status of children between the two sources. We then used a logistic regression model predicting probability of consistency between the two sources to estimate the probability of each record on the two administrative data sources being identified as being of Aboriginal and/or Torres Strait Islander origin in a survey. By summing these probabilities we produced model-adjusted time series of neonatal outcomes for Aboriginal and/or Torres Strait Islander births.

**Results:**

Compared to survey data, information based only on the two administrative data sources identified substantially fewer Aboriginal and/or Torres Strait Islander births. However, these births were not randomly distributed. Births of children identified as being of Aboriginal and/or Torres Strait Islander origin in the survey only were more likely to be living in urban areas, in less disadvantaged areas, and to have only one parent who identifies as being of Aboriginal and/or Torres Strait Islander origin, particularly the father. They were also more likely to have better health and wellbeing outcomes. Applying an adjustment model based on the linked survey data increased the estimated number of Aboriginal and/or Torres Strait Islander births in WA by around 25%, however this increase was accompanied by lower overall proportions of low birth weight and low gestational age babies.

**Conclusions:**

Record linkage of survey data to administrative data sets is useful to validate the quality of recording of demographic information in administrative data sources, and such information can be used to adjust for differential identification in administrative data.

## Background

The use of administrative data sets for measuring key indicators of health and wellbeing in populations has been expanding rapidly in recent years with the computerisation of administrative processes, advances in computer processing power that enable large datasets to be processed quickly, development of appropriate security and confidentiality protocols to liberate administrative data for analytic purposes, and the development of record linkage techniques and protocols that allow multiple administrative data sources to be combined. Administrative data sources have several key advantages over surveys and other specific data collection activities — the data is often routinely collected for entire populations, the additional cost of analysing administrative data is minimal, there is no additional burden placed on respondents, and the data can avoid the selection or participation biases common in direct data collection activities. Administrative data can be particularly useful for measuring outcomes in small population sub-groups where data collection activities would be very expensive given the high costs involved in screening or searching for the sub-population of interest. Such a case is the development of indicators of wellbeing among Aboriginal and Torres Strait Islander populations in Australia. Under the framework of the *National Indigenous Reform Agreement* ratified in 2008, the Council of Australian Governments (COAG) has established key targets and a reporting framework for measuring progress against these targets for *Closing the Gap in Indigenous Disadvantage*[1]. Many of these indicators are sourced from administrative data sets. Because of the high costs involved there are only a small number of other data collections that provide information on Aboriginal and Torres Strait Islander populations, such as the National Aboriginal and Torres Strait Islander Health Surveys [2] and National Aboriginal and Torres Strait Islander Social Surveys [3] conducted by the Australian Bureau of Statistics (ABS), and the Western Australian Aboriginal Child Health Survey (WAACHS) conducted by the Telethon Institute for Child Health Research in collaboration with the ABS [4-7].

Although administrative data sets have numerous advantages, there are some limitations that need to be considered when undertaking research and when developing indicator series from administrative data sources. The primary purpose of collecting administrative data is normally to support the administrative purpose of the agency undertaking the collection. This can affect the quality of some data items collected in administrative data sources. For example, demographic data of interest to researchers, but peripheral to the direct needs of the agency providing the service, may not always be rigorously pursued and carefully collected when included in administrative data collections. Agencies that collect administrative data have a legitimate hierarchy of needs for their data, and this can lead to some data items of particular interest to researchers being collected with lower attention to detail than would be the case in a specific research study.

One possible way to validate or possibly improve the quality of demographic data collected within administrative data systems is to compare the administrative data with information collected on the same people in other data collections. For instance, probabilistic record linkage of an administrative data source with other administrative data sources or surveys provides the opportunity to compare the reporting of demographic information in different settings and contexts.

We investigated the use of record linkage of administrative and survey data sources to:

 i) validate the quality of reporting of Aboriginal and/or Torres Strait Islander status collected in two administrative data collections—the WA Register of Births and the WA Midwives’ Notification System.

 ii) assess the potential impact of missing and inconsistently ascertained Aboriginal and/or Torres Strait Islander status on indicator series derived from these two administrative collections.

 iii) develop a model-based approach to make aggregate adjustments to indicator series derived from these two administrative collections.

We apply this technique specifically to the case of measures of neonatal status of Aboriginal and/or Torres Strait Islander births derived from the Western Australia (WA) Register of Births and the WA Midwives’ Notification System, although this approach could also potentially be used to adjust other variables within administrative data collections. The WA Register of Births is compiled electronically from paper birth registration forms filled in by the parents of each newborn within the days following the birth. The birth registration form requests the Indigenous status of both the mother and father of the child, although father information is optional. Significant amounts of missing data are recorded within the system (well in excess of 10% of birth registrations), as there is no in-person follow-up or verification of demographic information recorded on the forms. This information is supplemented in our analysis by the WA Midwives’ Notification System, an electronic database system recording neonatal measures for all births attended by a midwife in WA. This database includes an ethnicity field for the mother, one option of which is “Aboriginal or Torres Strait Islander”. This information is often collected during a pre-delivery interview with the expectant mother, but it is not known for what proportion of births the ethnicity question is not asked directly. Data from Midwives’ collections such as the WA Midwives’ Notification System are routinely used as the source of statistics on neonatal indicators for Aboriginal and/or Torres Strait Islander children in WA and elsewhere in Australia [8,9]. Several reports from New South Wales have concluded that improved identification of Aboriginal and/or Torres Strait Islander births can be achieved by combining midwives’ data with birth registrations [10-12].

In Australia a standard question has been developed for ascertaining Indigenous status [13]: 

"“Are you of Aboriginal or Torres Strait Islander origin?"

"(For persons of both Aboriginal and Torres Strait Islander origin, mark both ‘Yes’ boxes)

 □ No

 □ Yes, Aboriginal

 □ Yes, Torres Strait Islander”

"

Depending on the administrative process, the standard wording may not be consistently applied in all cases, or missing data may result when the question is not asked. Additionally, we recognise the right of Aboriginal and Torres Strait Islander people to self-identify, and to self-identify differently under different circumstances; this is another potential source of inconsistency in administrative data. This may reflect the perceived risks and benefits of identifying as being of Aboriginal and/or Torres Strait Islander origin, particularly if there is a belief that such identification may impact on the delivery of the relevant service.

As there are substantial missing data for Indigenous status of parents on the WA Register of Births, and as the WA Midwives’ Notification System only collects information about the mother’s status, there is significant potential for under-identification of Aboriginal and/or Torres Strait Islander infants. For instance, a child may be identified as being of Aboriginal and/or Torres Strait Islander origin if the father identifies as being of Aboriginal and/or Torres Strait Islander origin when the mother does not. This quite legitimate identification scenario would not be detected from the WA Midwives’ Notification System. We have linked these two administrative data sources to survey data from the WA Aboriginal Child Health Survey and the WA Child Health Survey to ascertain consistency of Indigenous identification between administrative and survey data sources.

## Methods

### Data sources

#### WA Register of Births

The birth registration form used in WA was changed in 1992 to collect information on the Indigenous status of both the mother and father using the standard question for assessing Indigenous status. The Birth Registration Form must be completed and lodged with the Registry within 60 days of birth. The registration form is supplied to the parents by the hospital or midwife who delivered the baby. Information supplied by the parents on the form is not normally verified against any other source.

#### WA Midwives' Notification System

The notification of case attended form has a field headed “Ethnic origin” within the section which records the demographic details of the mother [9]. There are two tick boxes, labelled “Caucasian” and “Aboriginal or Torres Strait Islander”, or a write-in box labelled “Other”. The guidelines to midwives describe the purpose of the field but give no specific instruction on how or when to ask for the information [14]. It is not compulsory for the mother to supply the requested information to the attending midwife. It is not known in what proportion of cases the field is populated without direct questioning of the mother.

#### Western Australian Aboriginal Child Health Survey (WAACHS)

The WAACHS was a large-scale state-wide survey of families with Aboriginal and/or Torres Strait Islander children aged 0–17 years. The field work was conducted between May 2000 and July 2002. Area-based stratified random sampling was used to select a random sample of approximately one in 6 Aboriginal and/or Torres Strait Islander children and young people in WA. Multi-stage sampling was used with the first stage of selection being 761 census collection districts (CDs). Each of these selected CDs was screened with interviewers going door to door searching for families with Aboriginal and/or Torres Strait Islander children. Some 166,287 dwellings were screened to identify 2,386 families of which 1,999 agreed to participate in the survey (84%). The survey collected information on 5,289 children aged 0–17 years living in these 1,999 families. All survey information was collected by face-to-face interviewing with the parents or carers in the home. Parents or carers were asked for consent to link survey information to records from the WA Register of Births and the WA Midwives’ Notification System, and 96% of carers in the survey gave this consent. Indigenous status of all members of the family was recorded within the household record form. The WAACHS survey methodology, content and processes have been extensively described in the four volumes of findings published from the survey [4-7].

#### WA Child Health Survey

The 1993 Western Australian Child Health Survey (WACHS) was an area-based stratified multi-stage survey of children aged 4–16 years in WA. There were 1,462 participating households including 2,736 children in scope of the survey, representing a response rate of 89%. All parents in the survey gave consent for their child’s survey information to be linked to birth and medical records. Full details of the survey methodology have been published elsewhere [15].

### Aboriginal and/or Torres Strait Islander births

The standard definition of Aboriginal and/or Torres Strait Islander status is based on self-identification. As self-identification is not feasible for infants, Aboriginal and/or Torres Strait Islander status for births is based on the parents’ identification of the child. The two administrative data sources considered here, the WA Register of Births and the WA Midwives’ Notification System, do not seek to specifically identify the Indigenous status of the child. The WA Register of Births can record the Indigenous status of both parents, while the WA Midwives’ Notification System records the Indigenous status of the mother only. As such these collections do not identify births consistently with the standard definition of Indigenous status. However, they are used as the source for time series data on neonatal outcomes for Aboriginal and/or Torres Strait Islander children as they are the key data sets available for producing these statistics.

### Record linkage

**Figure 1:**
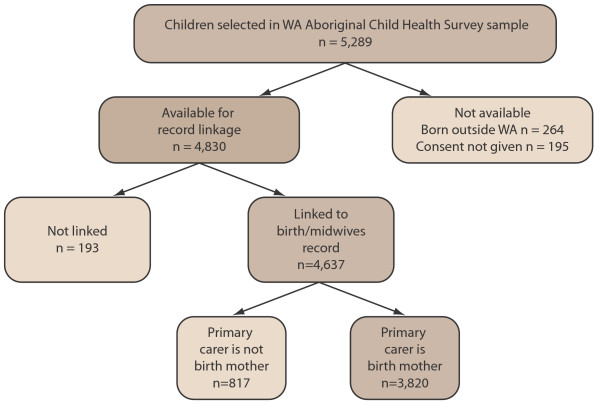
Linkage of WAACHS children with WA Register of Births and WA Midwives’ Notification System.

There were 5,289 children included in the WAACHS, and carers gave consent for record linkage in respect of 96% of these children. However, where the child’s carer at the time of the survey was not the birth mother of the child we were unable to ascertain the Indigenous status of the child’s birth mother during the survey. As both the WA Register of Births and the WA Midwives’ Notification System collect the Indigenous status of the mother, we restricted our comparison to those children whose carer at the time of the survey was the child’s birth mother. About 4% of children in the survey were born outside of WA and could not be linked to a WA birth registration. After accounting for these exclusions, 3,820 children were linked to their birth record on the WA Midwives’ Notification System (Figure 1).

### Analysis methods

Indigenous status of the mother as recorded on the WA Register of Births, the WA Midwives’ Notification System and as identified in the WAACHS were compared for all survey children whose records were linked to the WA Register of Births. In analysing WAACHS data, survey weights were used to estimate the total number of children within categories of Indigenous status of the mother as recorded in the various systems. The 5,289 children in the WAACHS sample were weighted up to 29,800 reflecting the true population of Aboriginal and/or Torres Strait Islander children aged 0–17 years at the time of the survey.

All of the children participating in the WAACHS were identified as being of Aboriginal and/or Torres Strait Islander origin by their parents or carers. This was the eligibility criterion for selection in the survey. The proportion of WAACHS children who would have been identified as being of Aboriginal and/or Torres Strait Islander origin using the parents’ Indigenous status on the birth registration form and Midwives’ Notification Form were calculated.

We investigated how the proportion of WAACHS children who would not have been identified as being of Aboriginal and/or Torres Strait Islander origin using either the WA Register of Births or the WA Midwives’ Notification System varied according to demographic characteristics. Characteristics investigated included: level of relative isolation of residential address, age of mother at time of birth, age and sex of child at time of the survey, family structure, and socio-economic status of Census Collection District of residence. Level of relative isolation was based on the ARIA++ classification developed by the National Key Centre for the Social Application of Geographic Information Systems at Adelaide University. The ARIA++ is an extension of the standard ARIA classification that is more useful for describing the circumstances of Aboriginal people living in remote areas as it includes more service centres, of smaller sizes, in calculating the remoteness scores. Full details of the level of relative isolation classification and its derivation have been published previously [4]. Family structure was assessed in the WAACHS by completing a full household record form and having interviewers draw a family tree for each family. This enabled identification of two-parent families, sole parent families, blended families and a range of extended family types. Full details of this derivation have been published previously [4]. Socio-economic status was based on a variant of the ABS Index of Relative Socio-Economic Disadvantage that was re-calculated for the survey omitting proportion of Aboriginal and/or Torres Strait Islander persons as a variable in the construction of the index [4].

We also investigated whether there were systematic differences in health and wellbeing outcomes between WAACHS children who were and weren’t able to be identified as being of Aboriginal and/or Torres Strait Islander origin using data from the WA Register of Births or the WA Midwives’ Notification System. To compare WAACHS children who were and were not identified as being of Aboriginal and/or Torres Strait Islander origin using these two administrative data sources we used weighted WAACHS survey data to calculate how the proportion of low birth weight babies (< 2500 grams), proportion of low gestational age babies (< 37 weeks gestation), proportion of children living in rented accommodation, and average test scores on two standardised tests from the British Ability Scales [6] — a test of visual-spatial reasoning and a vocabulary test — varied by consistency of identification between the data sources. Birth weight and gestational age were obtained from the WA Midwives’ Notification System, and proportion of children living in rented accommodation, and test scores were collected in the WAACHS.

### Adjusting administrative-based time series

We used data from the WAACHS children to fit a logistic regression model to predict the probability of being consistently identified as being of Aboriginal and/or Torres Strait Islander origin from both the WAACHS and the two administrative data sources. Explanatory variables included in the model were restricted to characteristics available on either the WA Register of Births or the WA Midwives’ Notification System: level of relative isolation of residential address, maternal age at time of birth, sex of child and marital status and family structure at time of birth of child.

As the WAACHS was specifically a survey of Aboriginal and/or Torres Strait Islander children, linkage to the survey is useful for identifying cases where a child would not be identified as being of Aboriginal and/or Torres Strait Islander origin on either the WA Register of Births or the WA Midwives’ Notification System but would be identified as such in the survey. However, to examine the rate of inconsistent identifications in the opposite direction, that is where a child would be identified as being of Aboriginal and/or Torres Strait Islander origin on either the WA Register of Births or the WA Midwives’ Notification System but would not be identified as such in a survey, we used data from the 1993 WA Child Health Survey. As the rate of inconsistency in this direction between survey and these administrative sources was very low for children identified as non-Indigenous in the survey, a constant probability was estimated for all children identified as non-Indigenous in the survey.

We applied the results of these models to estimate the probability of each child on the WA Register of Births or the WA Midwives’ Notification System data being identified in a survey as being of Aboriginal and/or Torres Strait Islander origin. We then calculated numbers of Aboriginal and/or Torres Strait Islander births in each year by summing these estimated probabilities. Thus the estimated probabilities are used as record weights for producing weighted counts. We also calculated original and adjusted time series of proportion of low birth weight births (< 2500 grams) and proportion of low gestational age births (<37 weeks). The original series were calculated by counting numbers of records per year. The adjusted series were calculated by summing the probabilities assigned to each record of the child being identified as being of Aboriginal and/or Torres Strait Islander origin in a survey. In addition to calculating annual figures, we smoothed the time series using LOESS smoothing [16].

Analysis was undertaken using SAS software. Due to the complex nature of the WAACHS sample design, which employed stratification and two levels of clustering, standard errors and confidence intervals for survey estimates were calculated using the ultimate cluster method of variance estimation [17], and regression models were fitted accounting for the sample design using Probability Weighted Iterative Generalised Least Squares [18] using special purpose SAS routines that were written for the survey. Full details of the estimation methods have been described previously [4].

### Ethical approval

The conduct of the WAACHS was approved by the Human Research Ethics Committee at Princess Margaret Hospital, and the Confidentiality of Health Information Committee at the Department of Health, WA. Ethical approval to link the survey data with records from the WA Register of Births and the WA Midwives’ Notification System was granted by the Confidentiality of Health Information Committee at the Department of Health, WA.

## Results

### Comparison of Indigenous status between the WAACHS and the WA Register of Births and the WA Midwives' Notification System

We compared the reported Indigenous status of the mother in the survey with that reported on the WA Midwives’ Notification System, and weighted figures are shown in Table 1. While all children in the survey were identified as being of Aboriginal and/or Torres Strait Islander origin, 15% of birth mothers did not identify as being of Aboriginal and/or Torres Strait Islander origin in the WAACHS, presumably cases where the father was of Aboriginal and/or Torres Strait Islander origin. Additionally for 8.5% of children whose mother identified as being of Aboriginal and/or Torres Strait Islander origin in the survey, the mother was identified as non-Indigenous on the WA Midwives’ Notification System. In contrast, less than half a percent of children had a mother who was identified as being of Aboriginal and/or Torres Strait Islander origin in the WA Midwives’ Notification System, but identified as non-Indigenous in the WAACHS.

**Table 1:** Aboriginal and/or Torres Strait Islander children whose carer is their birth mother: Indigenous status of mother as recorded in the WA Midwives’ Notification System compared with Indigenous status recorded in the WAACHS: weighted survey estimates, Western Australia (a) (b)

**Indigenous status of mother as recorded in the WAACHS**	**Indigenous status of mother as recorded in the WA Midwives’ Notification System**			
	**Aboriginal and/or Torres Strait Islander**	**Non-Indigenous**	**Not stated**	**Total**
Aboriginal and/or Torres Strait Islander	16,200	1,810	30	18,000
Non-Indigenous	90	2,940		3,030
Not stated	130	30		160
Total	16,400	4,780	30	21,200

(a) Estimates are rounded to 3 significant digits. Estimates may not sum to totals due to rounding.

(b) Estimates based on 3,820 WAACHS children whose primary carer was their mother and who were linked to their birth records.

Some WAACHS children were born prior to 1992, when the WA Register of Births started collecting Indigenous status of parents. Of those born in 1992 or later, the reported Indigenous status of the mother as compared with the WAACHS and the WA Midwives’ Notification System is shown in Table 2 using weighted survey data. There is substantial missing data from the self-reported Birth Registration forms, with 18% of children having the mother’s status missing. This proportion did not vary significantly over the period between 1992 and when the WAACHS field work was undertaken in 2000–2001. There was a higher proportion of missing data for father’s status on the WA Register of Births. Of 13,700 children, 7,420 had a father recorded as being of Aboriginal and/or Torres Strait Islander origin on the WA Register of Births, 1,710 had a father recorded as non-Indigenous, and 4,570 had father’s status not stated (33%). Of these 13,700 children, 8,990 had a mother recorded as being of Aboriginal and/or Torres Strait Islander origin, and an additional 1,590 had a father recorded as being of Aboriginal and/or Torres Strait Islander origin, so in total 77% of WAACHS children had one or both parents recorded as being of Aboriginal and/or Torres Strait Islander origin on the WA Register of Births.

**Table 2:** Aboriginal and/or Torres Strait Islander children born since 1992 whose carer is their birth mother: Indigenous status of mother as recorded in the WA Register of Births compared with Indigenous status recorded in the WAACHS and Indigenous status recorded in the WA Midwives’ Notification System: weighted survey estimates, Western Australia (a) (b)

**Indigenous status of mother as recorded in the WA Register of Births**	**Indigenous status of mother as recorded in the WAACHS**			
	**Aboriginal or Torres Strait Islander**	**Non-Indigenous**	**Not stated**	**Total**
Aboriginal or Torres Strait Islander	8810	130	60	8990
Non-Indigenous	460	1700	30	2190
Not stated	2230	280	10	2520
Total	11500	2100	100	13700
Indigenous status of mother as recorded in the WA Register of Births	Indigenous status of mother as recorded in the WA Midwives’ Notification System			
	Aboriginal or Torres Strait Islander	Non-Indigenous	Not stated	Total
Aboriginal or Torres Strait Islander	8360	600	20	8990
Non-Indigenous	200	1900	90	2190
Not stated	2060	420	40	2520
Total	10630	2920	150	13700

(a) Estimates are rounded to 3 significant digits. Estimates may not sum to totals due to rounding.

(b) Estimates based on 2,286 WAACHS children who were born from 1992 onwards, whose primary carer was their mother and who were linked to their birth records.

### Characteristics of children who would be identified differently between administrative and survey data sources

We found that there were some systematic differences between children who were identified consistently as being of Aboriginal and/or Torres Strait Islander origin in the WAACHS and the two administrative data sources compared to those who would not be identified as being of Aboriginal and/or Torres Strait Islander origin using only information from the WA Register of Births and the WA Midwives’ Notification System. There was a higher proportion of mothers identifying as being of Aboriginal and/or Torres Strait Islander origin in the WAACHS only who were living in areas of no or low relative isolation, and a substantially higher proportion of mothers identifying as non-Indigenous in the WAACHS, the WA Register of Births and the WA Midwives’ Notification System living in areas of no or low relative isolation. These latter cases are predominantly where the child is identified as being of Aboriginal and/or Torres Strait Islander origin because the father identifies as such (Table 3).

**Table 3:** Aboriginal and/or Torres Strait Islander children whose carer is their birth mother: Proportion of children by Indigenous status of mother in the WA Midwives’ Notification System and WAACHS: weighted survey estimates, Western Australia (a)

	**Proportion of children (%) (b)**	**Mother’s Indigenous status**		
		**Aboriginal and/or Torres Strait Islander in both WAACHS and Midwives’ Notification System (%) (c)**	**Aboriginal and/or Torres Strait Islander in WAACHS only (%) (c)**	**Non-Indigenous in both WAACHS and Midwives’ Notification System (%) (c)**
Level of relative isolation—				
None	36.9	63.1	13.6	23.3
Low	26.3	72.7	9.6	17.7
Moderate	20.5	88.1	3.6	8.3
High	9.2	96.1	1.8	2.1
Extreme	7.1	98.6	0.8	0.6
Relative socio-economic disadvantage of place of residence—				
Bottom 5%	24.2	86.6	6.8	6.7
5%-10%	12.7	79.9	7.5	12.6
10%-25%	26.5	74.1	9.6	16.3
25%-50%	26.9	71.5	8.7	19.9
Top 50%	9.7	65.5	10.9	23.6
Marital status—				
Married/de facto	66.5	75.1	9.3	15.6
Never married	31.6	79.8	6.7	13.5
Separated/ divorced/ widowed	1.5	61.6	11.8	26.6
Not stated	0.4	52.3	11.7	36.0

(a) Estimates based on 3,820 WAACHS children whose primary carer was their mother and who were linked to their birth records.

(b) Proportion of children in each category of interest, i.e. column percentages.

(c) Proportion of children by mother’s Indigenous status, i.e. row percentages.

Table 4 shows the comparison for a selection of health and wellbeing indicators as collected in the WAACHS for children who were consistently identified as being of Aboriginal and/or Torres Strait Islander origin in both the WAACHS and the registers, and those who would be identified as non-Indigenous using only data from the WA Register of Births and the WA Midwives’ Notification System. Children who would not be identified as being of Aboriginal and/or Torres Strait Islander origin using data from the two registers only were less likely to live in rented accommodation, had on average only half as many days absent from school in the last school year, and scored higher on both the word definitions test and visual-spatial reasoning tests administered in the survey. Additionally, when looking at neonatal measures for these children, a lower proportion of those who would be identified as non-Indigenous using only information from the WA Register of Births and the WA Midwives’ Notification System were born with birth weight less than 2500 grams or at gestational age of less than 37 weeks.

**Table 4:** Aboriginal and/or Torres Strait Islander children whose carer is their birth mother: Selected outcomes by Indigenous status of mother in the WA Midwives’ Notification System and WAACHS, Western Australia (a)

	**Mother’s Indigenous status**		
	**Aboriginal and/or Torres Strait Islander in both WAACHS and Midwives’ Notification System**	**Aboriginal and/or Torres Strait Islander in WAACHS only**	**Non-Indigenous in both WAACHS and Midwives’ Notification System**
Proportion living in rented accommodation (Per cent (95% CI))	78.5	59.4	58.5
	(75.6-81.1)	(50.7-68.2)	(49.2-67.9)
Average number of days absent from school (95% CI)	43.3	22.5	23.5
	(40.2-46.3)	(18.8-26.1)	(19.2-27.9)
Average test scores (centiles (95% CI))—			
Visual-spatial reasoning	45.8	49.7	52.0
	(44.7-47.0)	(47.7-51.7)	(49.6-54.4)
Vocabulary	32.0	40.7	38.7
	(30.8-33.3)	(38.4-43.0)	(34.5-42.8)
Proportion of low birth weight (<2500 grams)	13.3	10.5	9.9
	(12.0-14.6)	(7.0-14.0)	(7.3-12.5)
Proportion of low gestational age (<37 weeks) (Per cent (95% CI))	11.0	8.6	7.5
	(9.9-12.2)	(5.5-11.7)	(5.2-9.8)

(a) Estimates based on 3,820 WAACHS children whose primary carer was their mother and who were linked to their birth records.

### Inconsistent identification of non-Indigenous children in the 1993 WA child health survey

To estimate the probability that a child would be identified as being of Aboriginal and/or Torres Strait Islander origin from the WA Register of Births and the WA Midwives’ Notification system but would not be identified as such in a survey, we went back to the 1993 WA Child Health Survey. Although these data are now somewhat older than the WAACHS data, they are the most current sample of non-Indigenous children for whom Indigenous status has been assessed using face-to-face interviewing via the standard question and which have been linked to the WA Register of Births and the WA Midwives’ Notification System. As shown in Table 5, the proportion of non-Indigenous children as identified in a survey who would be identified as being of Aboriginal and/or Torres Strait Islander origin using these two administrative data sources is very low, around one half of one percent. This is consistent with the rate of which mothers of Aboriginal and/or Torres Strait Islander children who identified as non-Indigenous in the WAACHS were identified as being of Aboriginal and/or Torres Strait Islander origin in the WA Midwives’ Notification System.

**Table 5:** Children whose carer is their birth mother: Indigenous status of mother as recorded in the WA Midwives’ Notification System compared with Indigenous status recorded in the 1993 WA Child Health Survey, Western Australia (a)

**Indigenous status of mother as recorded in the Midwives’ Notification System**	**Indigenous status of mother as recorded in the 1993 WA Child Health Survey**			
	**Aboriginal and/or Torres Strait Islander**	**Non-Indigenous**	**Not stated**	**Total**
Aboriginal and/or Torres Strait Islander	22	25	2	49
Non-Indigenous	9	1,685	85	1,779
Not stated	0	26	3	29
Total	31	1,736	90	1,857

(a) unweighted sample counts.

### Time series of low birth weight and low gestational age babies

**Figure 2:**
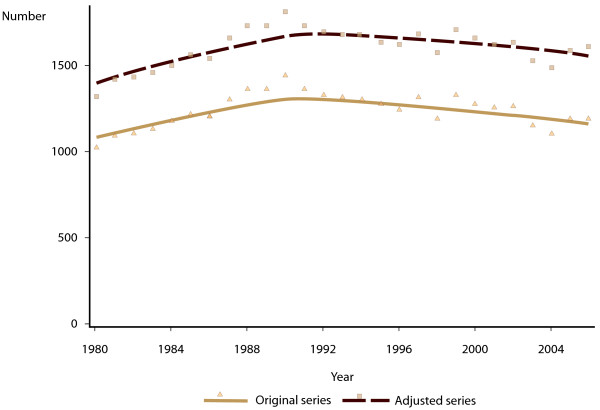
Number of Aboriginal and Torres Strait Islander births in Western Australia, 1980–2006, original register counts, and model-adjusted time series.

**Figure 3:**
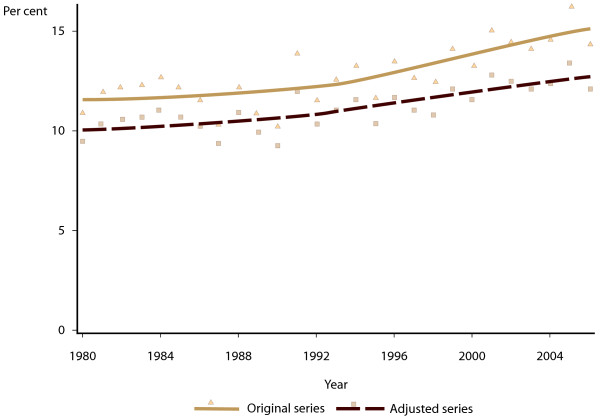
Proportion of Aboriginal and Torres Strait Islander births in Western Australia with birth weight less than 2500 grams, 1980–2006, original register counts, and model-adjusted time series.

**Figure 4:**
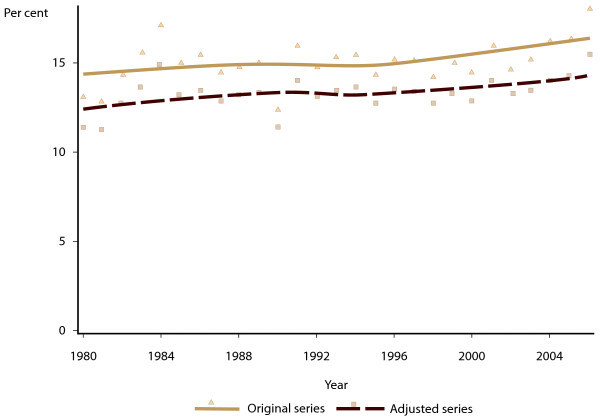
Proportion of Aboriginal and Torres Strait Islander births in Western Australia with gestational age less than 37 weeks, 1980–2006, original register counts, and model-adjusted time series.

Based on data from the 1993 WA Child Health Survey we estimated that the probability of being identified as being of Aboriginal and/or Torres Strait Islander origin on the WA Register of Births or the WA Midwives’ Notification System when the child would have been identified as non-Indigenous in a survey was 0.5%, constant for all Aboriginal and/or Torres Strait Islander children on the two registers. We estimated the probability of children being identified as non-Indigenous in the two administrative data sources who would have been identified as being of Aboriginal and/or Torres Strait Islander origin in a survey from the logistic regression model described previously. Original and model-adjusted numbers of births per year are shown in Figure 2. It can be seen that the model adjustment has resulted in an average increase in number of Aboriginal and/or Torres Strait Islander births of around 25% per year. Figure 3 shows the original and adjusted series for proportion of Aboriginal and/or Torres Strait Islander births that were of low birth weight, and Figure 4 shows the proportion that were low gestational age. In both cases, the increased number of births in the model adjusted series has resulted in a reduction in the proportion of Aboriginal and/or Torres Strait Islander births with adverse neonatal outcomes.

## Discussion

This study found substantial differences in identification of children between the survey data and the WA Register of Births and the WA Midwives’ Notification System. As the identifications in the survey were all made during face-to-face interviewing observing a standard protocol and using the standard question, these figures suggest that identifying Aboriginal and/or Torres Strait Islander births by using the Indigenous status of the mother on the WA Midwives’ Notification System would substantially under-identify Aboriginal and/or Torres Strait Islander births, both by missing cases where the father was identified as being of Aboriginal and/or Torres Strait Islander origin and the mother was not, and cases where the mother’s identification differed between the WA Midwives’ Notification System and the survey. Only 77% of the WAACHS children would have been identified as Aboriginal and/or Torres Strait Islander births using the mother’s reported status on the WA Midwives’ Notification System. Use of this field is the standard practice for deriving statistics on Aboriginal and/or Torres Strait Islander births in WA.

The process described in this paper for evaluating the consistency between identification of Indigenous status between a survey and an administrative data source can potentially be used for other demographic items collected in administrative data. For instance, socio-demographic indicators such as educational attainment, employment and occupation are collected in many administrative data sets. However, they are often not directly relevant to the administrative process, and are often of poor quality with substantial amounts of missing data. With the rapid expansion of the use of record linkage methodologies for undertaking research, validating the quality of fields such as these, with the potential to adjust analyses for observable patterns in inconsistent and under-recording has the potential to increase the quality of research undertaken using administrative data.

Other methods have been suggested for improving the quality of Indigenous identification in administrative data sets. The use of probabilistic record linkage to combine information from multiple data sets allows for the development of algorithmic approaches for filling in missing data on one data set with information from other data sets [19,20], or calculating a best practice indicator maximising available information across multiple data sets. Several studies have identified that under-identification of Aboriginal and Torres Strait Islander peoples in administrative data sets is non-randomly distributed, and methods to improve Indigenous identification in administrative data can result in both changes of counts of records, and changes in averages and other statistics derived from these records [21-24].

Identification of Aboriginal and/or Torres Strait Islander births directly from the WA Register of Births and the WA Midwives’ Notification System information in WA is not straight-forward. Relying on administrative data alone identifies substantially fewer Aboriginal and/or Torres Strait Islander births than is likely to be the case if parents were asked to directly identify the status of the child at the time of the birth. This is due to the significant proportion of missing Indigenous status for parents as recorded on the birth registration forms, and that the Midwives’ Notification Forms only record ethnicity of the mother, and even among the mothers not completely. Neither the WA Register of Births nor the WA Midwives’ Notification System is designed to identify the Indigenous status of infants. Both systems set out to record the status of one or both parents. However, it is common practice in the analysis of data from the WA Register of Births and the WA Midwives’ Notification System to derive the Indigenous status of the baby from the Indigenous status of the mother. This linkage study has shown that this practice will identify up to 25% fewer births as being of Aboriginal and/or Torres Strait Islander origin than otherwise might be the case.

However, the differences in identification status between the two systems are not randomly distributed. Children who are identified as being of Aboriginal and/or Torres Strait Islander origin consistently in both data sources were more likely to live in a regional, rural or remote area, more likely to live in areas of relative socio-economic disadvantage and were more likely to have worse outcomes on a range of measures of wellbeing. As a result, increasing the level of identification of Aboriginal and/or Torres Strait Islander births in the administrative birth data results in lower estimates of the proportion of low birth weight and low gestational age babies.

Applying the modelling approach used here results in lower overall prevalence estimates for poor neonatal outcomes, but the overall pattern among the time series is essentially preserved. This suggests that this approach could be useful in producing indicators designed to measure progress in closing gaps in Indigenous disadvantage. An alternative approach that has been used in other studies [19,20,25,26] is to link the administrative data source of interest to other administrative data sources which also include Indigenous status, and then use an algorithm to derive Indigenous status from combined data. This method has been used successfully, particularly in relation to improving the quality of mortality data [20,26]. It could potentially be used for birth data as well, if the data were being analysed sufficiently retrospectively for there to be sufficient time to have elapsed that the children would have had contact with other services where Indigenous status were collected.

The fact that children with consistent identification across both data sources have on average worse outcomes on all wellbeing measures used in this study reflects the fact that there is no single gap in wellbeing between Aboriginal and Torres Strait Islander peoples and non-Indigenous peoples. There are gradients in wellbeing outcomes among Aboriginal and Torres Strait Islander populations, as there are among non-Indigenous populations, by remoteness and by many other factors [4-7]. While the *Closing the Gap* indicators are useful aggregate measures to assess progress in meeting national goals, it is important that progress is not seen to be made solely by improving the way in which Aboriginal and Torres Strait Islander people are identified in source data sets, particularly if those improvements are driven by the inclusion of Aboriginal and/or Torres Strait Islander persons with clearly different socio-demographic profiles.

Where there are gradients in outcomes among Aboriginal and Torres Strait Islander people, studies should continue to be undertaken to measure and report on these differences. Progress in meeting national *Closing the Gap* targets will not necessarily mean that all Aboriginal and Torres Strait Islander people benefit equally from the improvements that are being made.

Indigenous status is determined by self-identification. Apart from differences in how or if the information is asked in a standard way, people may choose to identify differently in different situations, reflecting differing levels of cultural safety and appropriateness, and the perceived benefits or consequences of identifying as being of Aboriginal and/or Torres Strait Islander origin in each situation [24].

Standardising the process for collecting demographic data in administrative data collections can be difficult if data collection is part of the routine activity of an organisation and that activity itself varies in context between different operational centres. Smaller district and regional hospitals have different case loads and case mixes compared with facilities in the metropolitan area where there are several large facilities dedicated to the delivery of newborns. Smaller centres, particularly in remote areas, may also employ staff in differing roles, including community liaison roles which may result in greater likelihood of expectant mothers being known to staff. The proportion of the population that identifies as being of Aboriginal and/or Torres Strait Islander origin is larger in more remote areas, which may also affect the perceived relevance of asking for this information in administrative contexts. In settings where the number of mothers who identify as being of Aboriginal and/or Torres Strait Islander origin is very low, there may be less motivation for staff to take care with the collection of this data. The physical appearance of individuals could also affect administrative data collection, with staff possibly making inferences based on appearance, especially if they are uncomfortable asking the question.

The approach used here to validate the identification of Indigenous status using survey data is useful in quantifying overall trends and patterns in differences in identification. There was substantial under-identification of Aboriginal and/or Torres Strait Islander births just using information from the WA Register of Births and the WA Midwives’ Notification System, and this was particularly so for infants with a non-Indigenous mother and an Aboriginal and/or Torres Strait Islander father, and was also more common in less isolated areas. While this is useful information in understanding and interpreting data derived directly from administrative data sources, the method of estimating probability of identification in survey conditions is not specific enough to be able to accurately identify individual records where identification would have been different between survey and administrative sources. Thus the adjustments to the time series derived from the administrative sources based on using these probability weights should be considered as an aggregate adjustment to the time series. The technique is not sufficient in and of itself to identify all births for children who would be expected to subsequently identify as being of Aboriginal and/or Torres Strait Islander if a survey was conducted. Thus this approach is not appropriate if the end goal of a project is to analyse associations between Indigenous status and other outcomes at an individual level, such as via regression modelling using birth weight or gestational age as an outcome variable.

This study has some limitations. Patterns of Indigenous identification may change over time. Several studies investigating the quality of ascertainment of Indigenous status have reported that practices have improved over time in several registers [22-24]. However, no information is available as to how practices may have changed over time in respect of the WA Register of Births and the WA Midwives’ Notification System. If patterns change over time, since the WAACHS data was collected, the model used in this approach would be unable to reflect those changes.

Participation in the WAACHS was obviously limited to children who were alive at the time of the survey. As low birth weight and low gestational age babies may be at more risk of premature death it is possible that the exclusion of any chance of a second ascertainment of the Indigenous status of children who died prior to the survey could affect the results of our model. While premature mortality is significantly higher among Aboriginal and/or Torres Strait Islander babies and children [25], the small proportion of children who die at a young age suggests this is unlikely to have a major impact on the model fitted to these data.

The explanatory variables used in the models fitted to the data in this study were limited to those variables that are available on the administrative data sources, and thus available for use in calculating probability of identification in a survey setting. There may well be other factors that are relevant to describing the differences between those children who are identified consistently between the two sources and those who are not, which could not be captured in these data.

## Conclusions

Using mother’s status from the WA Midwives’ Notification System is standard practice for deriving statistics on Aboriginal and/or Torres Strait Islander births in WA. Compared with WAACHS data, this not only identifies substantially fewer Aboriginal and/or Torres Strait Islander births, but misses identifying births with systematically different characteristics. Births of children identified as being of Aboriginal and/or Torres Strait Islander origin in the WAACHS only were more likely to be living in urban areas, in less disadvantaged areas, and to have only one parent who identifies as being of Aboriginal and/or Torres Strait Islander origin, particularly the father. They were also more likely to have better health and wellbeing outcomes. Applying an adjustment model based on the survey data increased the estimated number of Aboriginal and/or Torres Strait Islander births in WA by around 25%, however this increase was accompanied by lower overall proportions of low birth weight and low gestational age babies.

Record linkage of survey data to administrative data can be a useful technique both to assess the quality of information recorded in administrative data systems, and as a means to improve the quality of statistics derived from administrative data sets. While this technique has been applied to the question of identifying Aboriginal and/or Torres Strait Islander births in birth records in WA, it has applicability to a wider range of demographic data recorded in administrative data sets. The conduct of population surveys and the linkage of administrative and survey data has the potential to significantly improve the quality of research based on linked administrative data sets.

## Competing interests

The authors declare that they have no competing interests.

## Authors' contributions

DL, DC and FM conceived the original idea for the study. All authors contributed to the development of the study methodology. DL acquired and analysed the data, and wrote the first draft of the manuscript. All authors edited the paper. All authors read and approved the final manuscript.

## Pre-publication history

The pre-publication history for this paper can be accessed here:

http://www.biomedcentral.com/1471-2288/12/90/prepub
